# QingBai decoction regulates intestinal permeability of dextran sulphate sodium‐induced colitis through the modulation of notch and NF‐κB signalling

**DOI:** 10.1111/cpr.12547

**Published:** 2019-01-18

**Authors:** Jun‐Chao Lin, Jie‐Qiong Wu, Fang Wang, Feng‐Ying Tang, Jia Sun, Bing Xu, Mingzuo Jiang, Yi Chu, Di Chen, Xiaowei Li, Song Su, Yujie Zhang, Nan Wu, Shaoqi Yang, Kaichun Wu, Jie Liang

**Affiliations:** ^1^ Department of Gastroenterology Second Affiliated Hospital of Shaanxi University of Traditional Chinese Medicine Shanxi China; ^2^ Department of Gastroenterology General Hospital of Ningxia Medical University Yinchuan China; ^3^ State Key Laboratory of Cancer Biology and Institute of Digestive Diseases Xijing Hospital, The Fourth Military Medical University Xi’an China; ^4^ Department of Gastroenterology NO. 307 Hospital of PLA Beijing China; ^5^ Laboratory of Tissue Engineering, Faculty of Life Science Northwest University Xi’an China

**Keywords:** intestinal permeability, MMP‐9, Mucin‐2, Notch, QingBai decoction, ulcerative colitis

## Abstract

**Objective:**

Chinese Herb QingBai decoction (QBD) has been approved affective in the treatment of IBD patients in clinic. However, the underlying mechanism remains unknown. We aim to investigate the effect of QBD on the mouse model of ulcerative colitis and its possible mechanism.

**Methods:**

C57/bL mice were given 5% DSS to induce colitis and were divided as QBD and mesalazine group. Weight, faeces and mental status were recorded each day and the histopathological changes (goblet cells etc) of the colon were observed after sacrificed. Fluorescein isothiocyanate‐dextran 4000 was measured to reflect the intestinal mucosal permeability. In addition, cell junction‐related proteins and possible signal pathways were investigated.

**Results:**

QingBai decoction could significantly alleviate the inflammation and the protection effect of colitis is comparable as those in mesalazine enema group. It was found that the permeability reduced significantly with QBD treatment vs the control group, while no significant difference between the mesalazine and QBD groups. QBD treatment could upregulate the expression of tight junction complex（ZO‐1, claudin‐1 and occludin）and muc‐2 expression. It significantly reduced the production and secretion of serials proinflammatory cytokines (IL‐1β, IL‐6, Kc and TNF‐α) compared with the control group. Meanwhile, NF‐κB and Notch pathways were regulated.

**Conclusion:**

QingBai decoction can effectively alleviate intestinal inflammation and mucosal barrier function in colitis mice, and the mechanism may be related to the inhibition of inflammatory cascade as well as enhanced mucus layer barrier and mechanical barrier function by NF‐κB and Notch signalling.

## 1 INTRODUCTION

Ulcerative colitis (UC) is a chronic, non‐specific intestinal inflammation, which is a refractory intestinal disease with the main pathological features of local ulcers and chronic inflammation of the colon. UC is difficult to cure, and it is prone to repeated attacks. Inducing and maintaining clinical remission and mucosal healing, improving quality of life, and preventing complications are considered to be the therapeutic targets of UC.^1^ At present, the main drugs for UC treatment are 5‐ASA drugs, steroid, immunosuppressants, biological preparations, etc. Previous clinical and experimental studies have proven that adjuvant traditional Chinese medicine treatments such as herbal medicine and acupuncture are beneficial to the relief of UC symptoms, with reliable efficacy, few side effects and low recurrence rate.^2^, ^3^

QingBai Decoction (QBD) can be used to treat UC based on traditional Chinese medicine (TCM) theory. Previous studies^4^ have found that QBD can effectively relieve intestinal symptoms such as diarrhoea, mucous bloody stool and fever for the treatment of mild‐to‐moderate active UC in clinic. The endoscopic examination after 8 weeks of treatment revealed that the mucosal healing was achieved, which improved the patient's quality of life.

Recent studies have shown that UC submucosal inflammation affects the integrity of the intestinal mucosal barrier structure, which leads to changes in permeability of the intestinal mucosa, and thus aggravates submucosal abnormal immune responses.^5^, ^6^ Therefore, objectively enhancing the function of the intestinal mucosal barrier may be a therapeutic solution for relieving intestinal inflammation. Our previous experiments have showed that QBD can improve the clinical symptoms caused by local inflammation, such as bloody purulent stool and local mucosal ulcers, but the mechanism is not yet clear. Therefore, in this study, the mechanism of QBD in UC treatment was studied from two aspects of inflammatory reaction and intestinal mucosal barrier function through the mouse model, and the mechanism of signal transduction to the pathway was discussed.

## 2 MATERIALS AND METHODS

### 2.1 Materials

DSS (AppliChem, Lot#：A3261‐0250 Germany); the six medicinal herbs of QBD raw powder, as shown in Table 1 (Pharmacy Department, The Second Hospital affiliated to Shanxi University of TCM, Xianyang, China) were decoting up 150 mL by traditional decocting method for enema； Mesalazine Enemas (V Vifor AG Zweigniederlassung Medichemie Ettingen， H20150127, Switzerland); faecal occult blood (FOB) (Baso Diagnostics Inc， Zhuhai， china); NF‐κB p65 antibody (Cell Signaling, #8242, Danvers, MA, USA); Phospho‐NF‐κB p65 antibody (Cell Signaling, #3039); p44/42 MAPK (ERK1/2) (antibody (Cell Signaling，#4695); Phospho‐p44/42 MAPK (ERK1/2) antibody (Cell Signaling, #4370); MMP‐9 antibody (Merck, AB19016, Burlington, MA, USA); Ki67 antibody (Merck, AB9260); Cleaved Caspase‐3 antibody (Cell Signaling, #9664); Caspase‐3 antibody (Cell Signaling, #9662); β‐Actin antibody (Cell Signaling,# 4970) Notch1 antibody (Cell Signaling, #3608s); ZO‐1 antibody (abcam, ab96587, Cambridge, MA, USA); Occludin antibody (abcam, ab 21632); claudin‐1 antibody (abcam, ab15098); primer synthesis (Takara Bio Inc, Dalian, China).

**Table 1:** Composition of QingBai decoction

Chinese name	Latin name	English name	Quantity in g
Da Qing Ye	Folium Isatidis	Indigowoad Leaf	12
Ban Lan Gen	Radix Isatidis	Indigowoad Root	20
Huang Bai	Cortex Phellodendri	Amur Corktree Bark	9
Ku Shen	Radix Sophorae Flavescentis	Lightyellow Sophora Root	20
Yi Ren	Semen Coicis	Coix Seed	30
Wu Zei Gu	Os SepieUae seu Sepiae	Cuttlebone	25

### 2.2 Colitis model construction and treatment

Wild‐type C57BL/6 mice were purchased from the Experimental Animal Center of fourth military Medical University [Certificate SCXK2012‐0007], twenty‐eight mice(weight, 21 ± 2 g) were randomly divided into four groups: the water group(treatment with water) (n = 8);the control group (n* = *8); the QBD group (n* = *8); and the mesalazine group (n* = *8). Except for the control group, Six‐ to eight‐week‐old mice were administered 5% DSS in drinking water for 5 days, then mice were killed 3 days after recovery with water. According to the conversion factor of experimental animals and clinical administration dose, the mice in QBD group and mesalazine group were administered with QBD enema (0.0195 mL/g per day, the suitable dose in our previous experiment, which show in supplementary information) or mesalazine enema (0.008 mL/g per day) by enemata for 8 day. The control group and the DSS group mice were administered normal saline by enemata. The colour, activity, faeces condition and weight were observed daily during modelling and drug treatment. FOB was tested and the severity of colitis was assessed daily using the disease activity index (DAI).

### 2.3 Real‐time RT‐PCR

Total RNA from colons was isolated with Takara Mini BEST Universal RNA Extraction Kit. RNA was denatured in the presence of an oligo dT primer and then reverse transcribed with Advantage RT‐for‐PCR Kit (Takara, Dalian, China [invested by Japan]). Quantitative PCR analyses were performed on Bio‐Rad CFX96 System using the SYBR Green PCR Master Mix (Takara). Samples were normalized to the control housekeeping gene GAPDH and reported according to the method of 2–ΔΔCt (ΔCt =ΔCttarget −ΔCtActin; ΔΔCt = ΔCtexpressing vector −ΔCtcontrol vector). Total RNA from colons was isolated with Takara Mini BEST Universal RNA Extraction Kit., and reverse transcription was performed using the Advantage RT‐for‐PCR Kit (Takara) according to the manufacturer's instructions. Quantitative PCR analyses were performed on Bio‐Rad CFX96 System using the SYBR Green PCR Master Mix (Takara). The cycling parameters were as follows: 95°C for 15 s, 55‐60°C for 15 s and 72°C for 15 s for 45 cycles. Samples were normalized to the control housekeeping gene ACTIN and reported according to the method of 2–ΔΔCt (ΔCt =ΔCttarget −ΔCt ACTIN; ΔΔCt = ΔCtexpressing vector −ΔCtcontrol vector). Table 2 shows the sequences of reverse and forward primers.

**Table 2:** Polymerase chain reaction primers' gene sequences

Target gene	Primer sequence	Product length in bp
ATOH1	Forward:AAATGTCGTATCTCTGCCTCTGGT C Reverse:AAGTACCCAATGCGGGTCTCAA	144
Hes‐1	Forward:AAAGACGGCCTCTGAGCAC Reverse:GGTGCTTCACAGTCATTTCCA	179
IL‐lb	Forward:TCCAGGATGAGGACATGAGCAC Reverse:GAACGTCACACACCAGCAGGTTA	105
TNF‐α	Forward: ACTCCAGGCGGTGCCTATGT Reverse:GTGAGGGTCTGGGCCATAGAA	160
IL‐6	Forward:CCACTTCACAAGTCGGAGGCTTA Reverse:TGCAAGTGCATCATCGTTGTTC	113
Kc	Forward:TGCACCCAAACCGAAGTC Reverse:GTCAGAAGCCAGCGTTCACC	175
Actin	Forward: TTTTCCAGCCTTCCTTCTTGGGTAT Reverse:CTGTGTTGGCATAGAGGTCTTTACG	111

### 2.4 Immunohistochemistry analysis

Briefly, paraffin‐embedded slides were deparaffinized, and antigen unmasking was carried out by microwave heating in a citrate buffer for 20 min. After washing with PBS, slides were incubated with 3% H2O2 and then goat serum for 10 min and 15 min at room temperature, respectively. Primary antibodies were added to slides for incubating at 4°C overnight. Biotinylated secondary anti‐rabbit antibodies were added and incubated at room temperature for 15 min. After 15 min with streptavidin‐HRP, sections were stained with DAB substrate for 5 min. According to previous reports, the score of immunostaining intensity was performed.

### 2.5 Western blot analysis

Tissue was extracted using RIPA lysis buffer with the protease inhibitor phenylmethane sulfonyl fluoride (Merck Millipore, Burlington, MA, USA). Protein concentration was measured using the BCA protein assay kit (Beyotime Biotechnology) according to the manufacturer's instructions. Equivalent amounts of protein (20 μg) were separated on SDS PAGE gel and then transferred onto 0.45 μm PVDF membranes (Millipore, Billeria, MA, USA) according to the standard protocols. Membranes were blocked in 5% milk in TBST buffer for 1 h at room temperature, followed by incubation with primary antibodies at 4°C overnight. After incubation with a secondary antibody for 1 h at room temperature, proteins were detected using ECL reagent (Millipore). Three independent replicates were conducted for each.

### 2.6 Statistical analysis

One‐way ANOVA was used to analyse the data expressed as mean ± SD for comparison between multiple groups and least significant difference *t *test for internal group comparison. All statistical analyses were performed using SPSS version 19 (Standford, CA, USA). The threshold of statistical significance was set to *P < *0.05. GraphPad Prism version 6.02 was used to generate histograms.

## 3 RESULT

### 3.1 QingBai decoction promotes recovery of DSS‐induced colitis in mice

**Figure 1:**
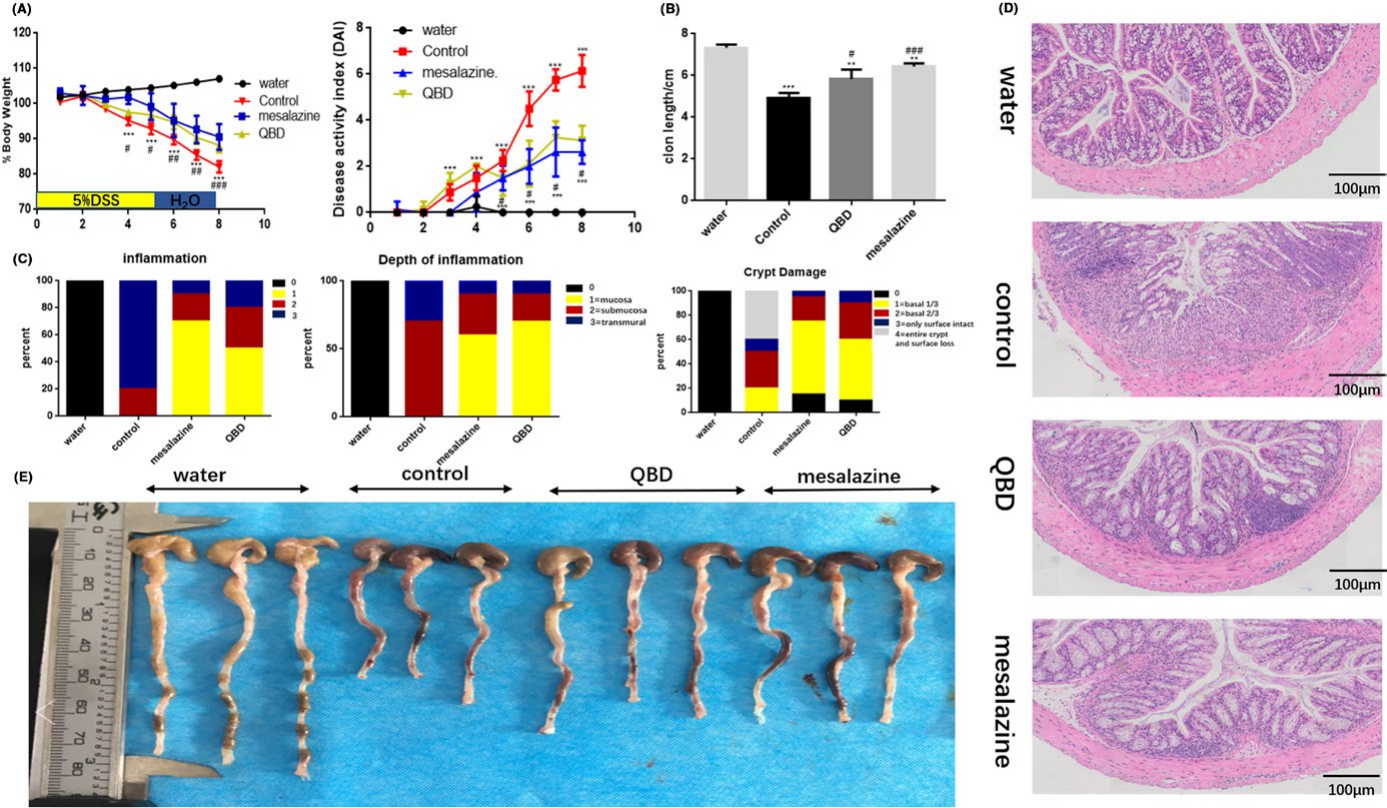
QBD promotes recovery of DSS‐induced colitis in mice. Except the water group， mice in other groups were given DSS (5% w/v) for 5 days and the mice in the QBD and were performed enema with QBD or mesalazine, respectively. (A) Body weight change (the change of body weight in each group of mice was compared with the body weight in the same group of mice on the 1st day); Disease activity index（QBD group vs control group）； and (B) Length of colon (from the appendix to the anus); (C) Histopathologic scoring of the inflammation, depth of inflammation and crypt damage. Data are represented as the percentage of mice per group with the indicated score; (D) Histologic images of mice colon (H and E, magnification ×100). A: water group; B: control group; C: QBD group; D: SASP group; (E) Macroscopic appearance. *P < 0.05 vs water group **P < 0.01 vs water group ***P <0.001 vs water group, ^#^ *P *< 0.05 vs control group ^##^ *P *< 0.01 vs control group ^###^ *P *< 0.001 vs control group. Water group: treatment with water; SASP group: treatment with mesalazine; Control group: treatment with Dextran sulphate sodium; QBD: treatment with QingBai decoction

To induce acute colitis, mice were treated with 5% DSS for 5 days. Increased epithelial injury and production of inflammatory cytokines are known characteristics of this mouse model. To evaluate the treatment effect of QBD on DSS‐induced colon epithelial injury and colitis, we further determined whether QBD have the ability to ameliorate established colitis. On the 4th day of DSS‐administration, body weight loss was observed significantly in the control group compared to QBD and mesalazine groups (*P* < 0.001) (Figure 1A), which continued until day‐8 of DSS‐administration. QBD and mesalazine groups showed a significant decrease in the DAI scores (*P* < 0.001), compared with control group. (Figure 1A). QBD and mesalazine treatment significantly rescued DSS‐induced colon shortening compared to the model group (*P* < 0.05) (Figure 1B), However, no statistically significant difference was found between these two groups (*P* > 0.05). We also observed less congestion and oedema in the colon of QBD and mesalazine groups compared to the control groups (Figure 1E). The H&E staining showed epithelial crypt damage and severe mucosal inflammation in the control group, which were less severe in QBD and mesalazine groups (Figure 1D). Apart from H&E staining, a significant decrease of histopathological scoring in QBD and mesalazine groups was detected vs* the* control group represented by the inflammation, depth of inflammation and crypt damage (Figure 1C). These data suggest that QBD exerts therapeutic effects on severe colitis associated with intestinal epithelial cell injury probably.

### 3.2 QingBai decoction inhibits apoptosis and promotes proliferation in the colon of DSS‐treated mice

**Figure 2:**
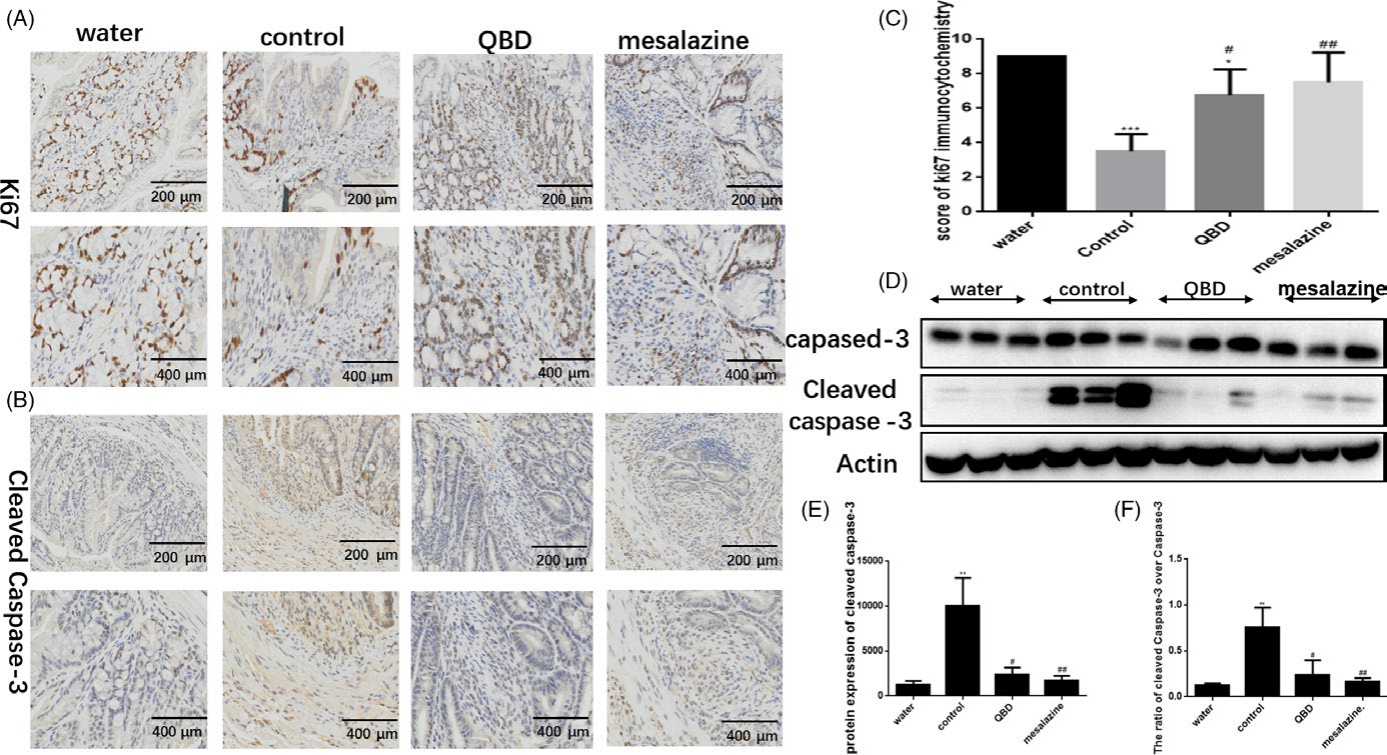
QBD inhibits apoptosis and promotes proliferation in the colon of DSS‐treated mice. Apoptosis and proliferation in the colon from both groups (day‐8) were examined (A) Immunohistochemistry staining for Ki67; and (B) Immunohistochemistry staining for active caspase‐3; (C) The score of Ki67 immunohistochemistry; (D) Representative western blots of total caspase‐3 and active caspase‐3; (E) Quantitative analysis of active caspase‐3. *P < 0.05 vs water group **P < 0.01 vs water group ***P < 0.001 vs water group, ^#^ *P *< 0.05 vs control group ^##^ *P *< 0.01 vs control group ^###^ *P *<0.001 vs control group. Water group: treatment with water; SASP group: treatment with mesalazine; Control group: treatment with Dextran sulphate sodium; QBD: treatment with QingBai decoction

The previous reports believed that DSS is directly toxic to gut epithelial cells of the basal crypts, which increased intestinal epithelial cell apoptosis. The apoptotic rate of intestinal epithelial cells is significantly increased in active UC. Another study showed that a normalization of the apoptotic rate after anti‐TNF treatment. Therefore, the proliferation and apoptosis were detected using immunohistochemical analysis to determine Ki67 and active caspase‐3 positive cells, and immunoblot analysis to determine the expression of active caspase‐3 in the colon. The number of Ki67 positive cells decreased while active caspase‐3 positive cells increased in the control group compared with QBD and mesalazine groups (Figure 2A,B). The score of Ki67 immunohistochemistry increased significantly in the QBD and mesalazine groups compared to control group（*P* < 0.05）(Figure 2C). At the same time, the control groups demonstrated significant increased active caspase‐3 expression compared to QBD and mesalazine groups(*P* < 0.05) (Figure 2D,E). Then the ratio of cleaved caspase‐3 over caspase‐3 was significant difference between control group and QBD group (Figure 2F). We found no significant difference in the apoptosis and proliferation between the QBD and mesalazine groups. Combined, these data suggest that QBD probably regulates apoptosis and proliferation in DSS‐induced colitis, which may decrease apoptotic rate to promote recovery of injured epithelial cells.

### 3.3 QingBai decoction attenuate DSS‐induced epithelial permeability through regulates colonic epithelial cell function

**Figure 3:**
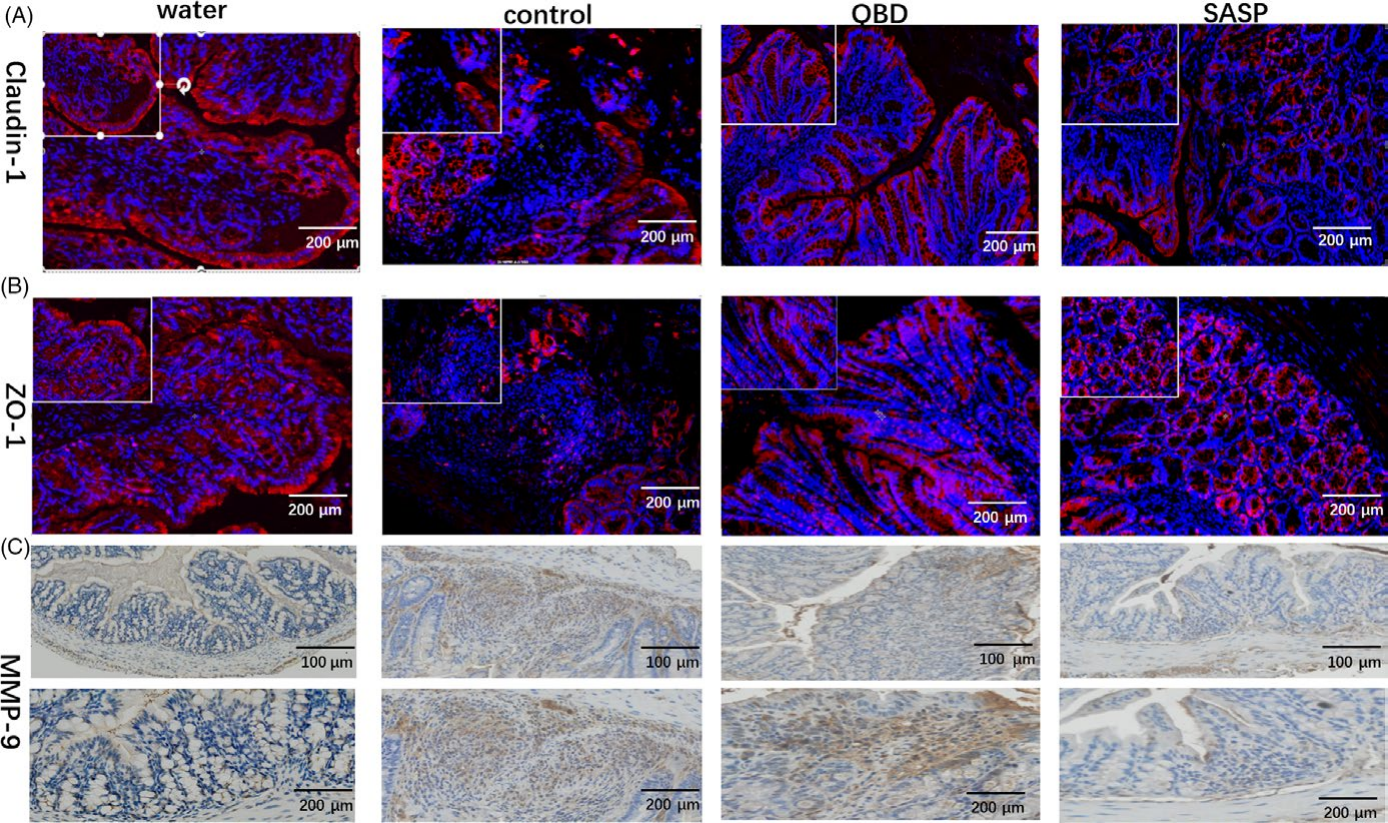
QBD ameliorates the damage of junctional complex induced by DSS through regulates the expression of MMP‐9. QBD preserves intestinal barrier function in DSS‐treated mice. Paraffin‐embedded colon tissues were used to determine zonula occludens‐1 (ZO‐1) and claudin‐1 distribution by immunohistochemistry using an anti‐ZO‐1 antibody and anti‐claudin‐1 antibody， then FITC‐labelled secondary antibody were used and observed by fluorescence microscopy (red staining). Nuclei were stained with 4,6‐diamidino‐2‐phenylindole (DAPI; blue staining) (*A*). The distribution of ZO‐1 in the colon from both groups; (B). The distribution of claudin‐1 in the colon from both groups (C). Immunohistochemistry staining for MMP‐9 in colon. *P < 0.05 vs water group **P < 0.01 vs water group ***P < 0.001 vs water group, ^#^ *P *< 0.05 vs control group ^##^ *P *< 0.01 vs control group ^###^ *P *<0.001 vs control group. Water group: treatment with water; SASP group: treatment with mesalazine; Control group: treatment with Dextran sulphate sodium; QBD: treatment with QingBai decoction

**Figure 4:**
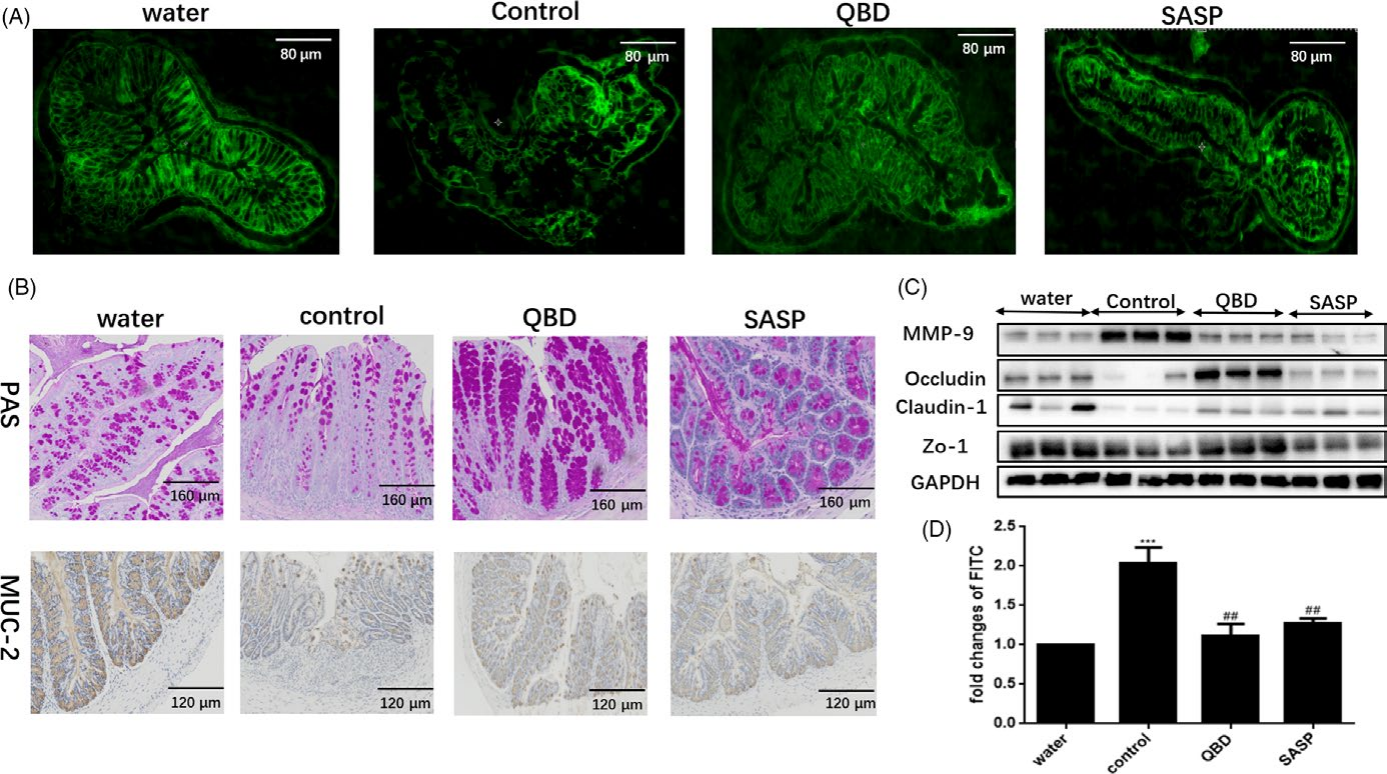
QBD attenuate DSS‐induced epithelial permeability through enhances the function of mucus layer barrier and mechanical barrier. QBD preserves epithelial permeability in DSS‐treated mice.(A) submucosal infiltration of FITC‐dextran was examined 4 h after administration. The frozen sections of colons observed by fluorescence microscopy;(B) Immunohistochemistry staining for PAS and Muc‐2 in colon;(C) the expression of MMP‐9, ZO‐1, claudin‐1 and occludin were detected by Western blot analysis; (D) the fold changes of FITC from each groups. *P < 0.05 vs water group **P < 0.01 vs water group ***P < 0.001 vs water group, ^#^ *P *<0.05 vs control group ^##^ *P *<0.01 vs control group ^###^ *P *<0.001 vs control group. Water group: treatment with water; SASP group: treatment with mesalazine; Control group: treatment with Dextran sulphate sodium; QBD: treatment with QingBai decoction

Extracellular mucus and tight junctions are two essential constituents for mucosal barrier, which against detrimental substances in the intestinal lumen. DSS model would not only regulate intestinal epithelial cell apoptosis, but also affect the integrity of the mucosal barrier. The damage of intestinal permeability may due to dysregulation of either the mucus or the epithelial junctional complex, which enhances susceptibility to colitis. The changes in epithelial permeability were detected by FITC‐Dextran experiment*.* Compared with the control group, the permeability of the colon mucosa reduced significantly in QBD and mesalazine groups（*P* < 0.05） (Figure 4D), the injured of mucosa was also observed by fluorescence microscopy in frozen sections of colons (Figure 4A). In addition, the number of muc‐2 and goblet cells positive cells increased obviously in colon after the treatment of QBD or mesalazine (Figure 4B). As marker of tight junction structure, the distribution and expression of ZO‐1 and claudin‐1 were detected using immunostaining and immunoblotting, respectively. The redistribution and depletion of these two proteins caused by DSS were reversed by QBD and mesalazine (Figure 3A,B). We also found that ZO‐1, claudin‐1 and occludin protein levels increased significantly in the QBD and mesalazine groups vs control group（*P* < 0.05）(Figure 4C). Therefore, we determined the expression of active MMP‐9 by immunoblotting and immunostaining. Immunoblot analysis demonstrated decreased expression of active MMP‐9 in the QBD and mesalazine group vs control group (*P* < 0.05) Figure 3C and 4C）. Thus， the protective roles of QBD in the intestinal epithelium may attenuate epithelial permeability in DSS‐induced colitis.

### 3.4 QingBai decoction inhibits Notch signalling in the colon of DSS‐treated mice

**Figure 5:**
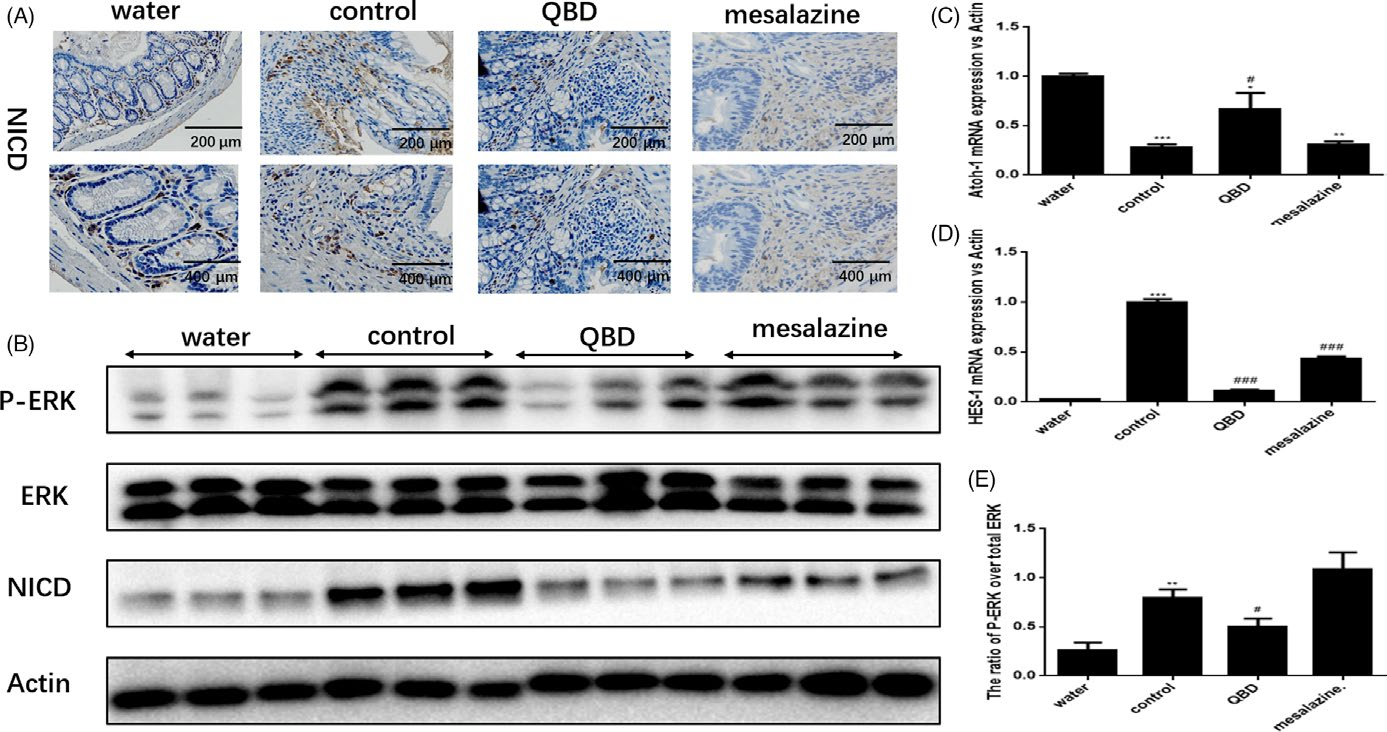
QBD inhibits Notch signalling in the colon of DSS‐treated mice. (A) Immunohistochemistry staining for NICD in colon tissue samples; (B) Immunoblot analysis to determine the expression of NICD, p‐ERK1/2 and total ERK1/2 in colon tissue samples; (C), (D) real‐time PCR for Hes‐1 and ATOH1 in colon tissue of each groups. *P <0.05 vs water group **P <0.01 vs water group ***P <0.001 vs water group, ^#^ *P *< 0.05 vs control group ^##^ *P *< 0.01 vs control group ^###^ *P *< 0.001 vs control group. Water group: treatment with water; SASP group: treatment with mesalazine; Control group:treatment with Dextran sulphate sodium; QBD: treatment with QingBai decoction

The Notch signalling pathway plays a critical role of the intestinal epithelial cell fate determination,^7^ which induces transcription of a number of genes including Hes‐1 to regulate goblet cells and Muc‐2 expression. Previous studies have shown that dysregulation of the differentiation system for prompt intestinal epithelial cell formation induces the pathology of intestinal diseases as ulcerative colitis (UC).^8^Therefore, we examined the status of Notch signalling (using immunostaining and immunoblotting) and the change in mRNA of Hes‐1 and ATOH1(using real‐time qPCR analysis). In agreement with previous reports, we found that the protein expression of NICD, p‐ERK, ERK and the ratio of p‐ERK over total ERK increased significantly in the control group (Figure 5B,E). The immunostaining showed that NICD positive cells reduce in colon submucosal Inflammatory after the treatment of QBD (Figure 5A). Moreover, the mRNA of Hes‐1 decreased significantly while the mRNA of ATOH1 increased significantly in QBD group vs control group (*P* < 0.01) (Figure 5C,D). After the treatment of QBD, the protein level of NICD and p‐ERK decreased significantly in QBD group compared to control group(*P* < 0.05). To further demonstrate that QBD prevents the loss of muc‐2 expression in DSS mice by inhibiting Notch signalling. We used the inhibitor of Notch signalling in vitro experiment, then we found that the effect of QBD on muc‐2 is less efficient after Notch signalling was blocked（show in supplementary information）. Thus, our results suggested that QBD prevent the loss of muc‐2 expression *in DSS‐treated mice* by *inhibiting* Notch signalling probably.

### 3.5 QingBai decoction inhibited the NF‐κB pathway, regulated the cytokine expression of IL‐1β, IL‐6, Kc and TNF‐α mRNA levels in colon tissue

**Figure 6:**
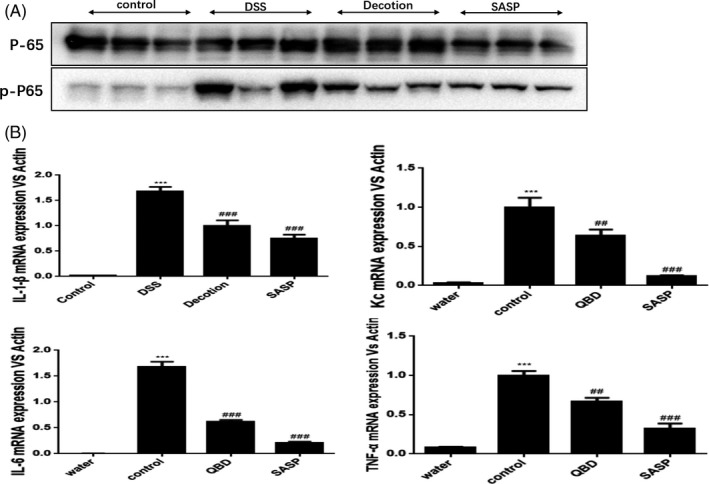
QBD inhibited the NF‐κB pathway, regulated the cytokine expression of IL‐1β, IL‐6, Kc and TNF‐α mRNA levels in colon tissue. (A) Immunoblot analysis to determine the expression of p‐p65 and total p65 in colon tissue samples;(B) real‐time PCR for IL‐1β， IL‐6， Kc and TNF‐α in colon tissue of each groups. *P < 0.05 vs water group **P < 0.01 vs water group ***P < 0.001 vs water group, ^#^ *P *< 0.05 vs control group ^##^ *P *<0.01 vs control group ^###^ *P *< 0.001 vs control group. Water group: treatment with water; SASP group: treatment with mesalazine; Control group: treatment with Dextran sulphate sodium; QBD: treatment with QingBai decoction

NF‐κB served as a major regulator of innate immunity and inflammatory responses^9^， which regulates the production of proinflammatory cytokine. The effects of QBD in the DSS‐induced colitis model were further studied. We compared mRNA expression levels of the key inflammatory cytokines, TNF‐α, IL‐1β, IL‐6 and chemokine KC/CXCL1 using q‐PCR. The control group showed significantly increased cytokine production including TNF‐α, IL‐1β, IL‐6 and chemokine KC after DSS induction (*P* < 0.01) (Figure 6B). Compared with control group, a significantly reduced cytokine production including TNF‐α(*P* < 0.01), IL‐1β(*P* < 0.001), IL‐6(*P* < 0.001), and chemokine KC (*P* < 0.01) was observed in QBD group. We also detected the protein expression of P‐65 and P‐P65 using immunoblotting. The QBD and mesalazine group showed significantly decreased in the protein level of P‐P65 compared with control group (*P* < 0.05) (Figure 6A), while it was no significant difference for QBD and mesalazine groups. These data suggest that regulation of signalling pathways involved in proinflammatory cytokine production may be another mechanism by which QBD inhibits proinflammatory responses in DSS‐induced colitis model.

## 4 DISCUSSION

Ulcerative colitis is a chronic inflammatory disease， which results from a combination of genetic susceptibility,dysregulated mucosal barrier, and dysregulated immune response. Although the main drugs for UC treatment have a beneficial effect， there are some limitations of medical treatment such as severe side effects， long course of treatment and unsatisfied curative effect. It is necessary to seek out alternative or supplementary therapy for UC. Chinese Herb QingBai decoction （QBD）can be used to treat UC based on the basic theory of TCM (traditional chinese medicine), which is a full‐featured decoction. Modern studies have revealed that the presence of oxymatrine in Sophora flavescens significantly improves the degree of inflammation in a mouse model of DSS‐induced colitis, which is achieved because PI3K/Akt signalling pathway can play a role of anti‐inflammation and suppressing differentiation of Th1 and Th17 cells.^10^ Indirubin, an active ingredient in isatis root and folium isatidis, can significantly reduce intestinal inflammation in mice with DSS‐induced colitis by inhibiting the NF‐κB signalling pathway and inhibiting the infiltration of CD4^+^ T cells in the colonic mucosa.^11^ Berberine, the active ingredient in phellodendron, can protect tight junction proteins as well as relieve inflammatory responses in intestinal epithelium and macrophages in a mouse model of DSS‐induced colitis^12^

In this study， we reported a function of QBD in the regulation ofintestinal permeability and mucosal inflammation using the DSS‐induced colitis mice model. Recent studies have found that changes in intestinal permeability play an important role in the progression of intestinal inflammation.^6^ The function of the mucosal barrier is mainly composed of two parts: the mucus layer barrier and mechanical barrier formed by the tight junction between the intestinal epithelial cells. The mucus layer is mainly composed of mucoprotein secreted by the goblet cells and immunologic active substances secreted by other mature intestinal epithelial cells, which is capable of sequestering pathogenic factors in the outermost mucus and discharging them into the intestinal lumen as the mucus layer continuously updates.^13^ The tight junction between epithelial cells is composed of transmembrane proteins (occludin, claudins, junctional adhesion molecules) and scaffold proteins (ZO‐1, ZO‐2, ZO‐3, etc) that connect the transmembrane proteins to the cytoskeletons, especially the tight junction at the top of the cells plays a major role in regulating mucosal permeability.^14^, ^15^

Importantly, Notch signalling pathway plays an important role in maintaining the proliferation and differentiation of colonic epithelium.^7^ The activated fragment of Notch (NICD) can activate the transcription factor to induce the differentiation direction of intestinal epithelial cells after entering nuclei. Notably, aberrant expression of the Notch signalling pathway in the UC progression can lead to an increased expression of the transcription factor Hes‐1 (which inhibits the transcription factor ATOH1) to inhibit the differentiation of intestinal epithelial cells into goblet cells and thereby weaken the mucus barrier.^8^, ^16^, ^17^ Consequently, Notch activation and muc‐2/goblet cell depletion was observed in colitis model, which is consistent with previous reports. Our data suggest that QBD probably prevent the loss of muc‐2 expression to strengthen mucus barrier *through inhibiting* p‐ERK and Notch signalling. In addition, Matrix metalloproteinases (Mmps) are zn^2+^‐dependent endopeptidases that can solubilize and regulate ECM balance under physiological conditions. Dysregulation of MMPs expression in pathological states can amplify and prolong inflammation, as well as induce intestinal injury and inflammation. Moreover, MMPs are also involved in regulating activation of inflammatory factors, migration and differentiation of neutrophils, as well as degradation of tight junction proteins between cells.^18^ We using qPCR analysis to test the expression of MMPs known to be regulated in IBD, and show that MMP‐9 was down regulated after the QBD treatment. Previous studies have demonstrated that MMP‐9 can aggravate intestinal inflammation and destroy intestinal epithelial cells by regulating the activity of IL‐8 in UC mouse model.^19^ It has been reported that MMP‐9 mediates the direct action of MLCK on tight junction proteins, which results in the activation of myosins and subsequent contraction of peripheral cytoskeleton and mechanical opening of the TJ barrier.^20^ Our data show QBD decreased the permeability by *inhibiting* the expression of MMP‐9, which may destroy tight junction complex and intestinal epithelial cells.

The abnormal activation of the NF‐κB signalling pathway in the mucosal layer can lead to increased levels of inflammatory cytokines such as TNF‐α, IL‐1β and IL‐6.^9^ which induces an inflammatory cascade, and a large number of aggregated neutrophils can induce a series of pathological changes such as intestinal epithelial cell injury, crypt abscesses and small‐vessel vasculitis.^21^ Meanwhile, Intestinal permeability can be increased physiologically in response to pathologically by mucosal immune cells and cytokines.^22^ Our studies using qPCR analysis showed that the expression of proinflammatory cytokine known to be upregulated by NF‐κB signalling is decreased in QBD group. Moreover, the increased intestinal epithelial cell apoptosis caused by severe inflammatory may destroy tight junction proteins, and lead to a decreased barrier function and increased permeability.^23^, ^24^ While activated NF‐κB can also induce apoptosis of intestinal epithelial cells by mediating caspase‐3 activation, thereby to increase the permeability of intestinal mucosa.^9^, ^25^ We observed the expression of active caspase‐3 is significantly decreased in QBD group (compared to the control group). Combined, we suggest that QBD decreased the intestinal permeability by inhibiting NF‐κB signalling to regulate mucosal inflammation.

Taken together, central outcome from our current study is that QBD alleviated mucosal inflammation in colitis model may through inhibiting NF‐κB signalling and Notch activity to rescue the integrity of intestinal permeability from damage.

## CONFLICT OF INTEREST

The authors declare no conflict of interest.
