# Supplementary material for: QingBai decoction regulates intestinal permeability of dextran sulphate sodium‐induced colitis through the modulation of notch and NF‐κB signalling
Source: Cell Prolif. 2019 Jan 18;52(2):e12547. doi: 10.1111/cpr.12547 (PMC6496276; doi:10.1111/cpr.12547)
Supplement: Supplementary file 1 [file CPR-52-e12547-s001.docx]

Dear Dr Professor Qi Zhou:

Thank you very much for your decision letter on 14-Aug-2018 regarding our manuscript entitled " QingBai decoction regulates intestinal permeability of dextran sulfate sodium-induced colitis through the Modulation of Notch and NF-kb Signaling ".

We sincerely appreciate your kind advices, and a careful revision of this manuscript has been finished according to the suggestions. We evaluated the manuscript and revised it according to the comments. Point-by-point responses to the reviewers’ comments are listed below in this letter.

We hope that this well edited version of our manuscript is now acceptable for publication in Cell Proliferation.

Look forward to hearing from you soon.

Sincerely,

jie-Liang

State Key Laboratory of Cancer Biology and Xijing Hospital of Digestive Diseases, Fourth Military Medical University, Xi’an, 710032, China

Email: jie-concise@hotmail.com

Answers to reviewers:

Reviewer #1:

Major issues:

1）. Why you used one dose of QBD to explore its effect on experimental colitis? Normally, a pharmacological study would use at least two or more doses to observe the efficacy of tested compounds or formula (?).

**Answer:**

Thanks for your kindly comments. In our previous study, We found that the high dose of QBD probably have better ability to ameliorate established colitis than other doses and the macrography of colon in each groups as follow. So we take this dose to explore its effect on experimental colitis.

The mice were randomly divided into six groups (n = 4per group): DSS group (the model group), DSS + the low dose（0.0065 ml/g per day） group, DSS + the middle dose (0.013 ml/g per day) group, DSS +the high dose(0.0195ml/g per day) group, DSS + double dose of high dose (0.039ml/g per day) group and mesalazine group（0.008ml/g per day）in our previous group. every Six- to eight -wk-old mice were administered 5% DSS in drinking water for 5 days, then mice were killed 3 days after recovery with water. According to the conversion factor of experimental animals and clinical administration dose, the mice in the low dose 、the middle dose 、the high dose and double dose of high dose group and mesalazine group were administered with QBD enema or mesalazine enema by enemata for 8 d. Then we sacrificed both mice and we found that the effect of QBD was dose dependent. So we choice the high dose as the to explore its effect on experimental colitis

2. Primers IL-17 and GAPDH were revealed in manuscript but the related-data was not showed in figures, and the primer of ACTIN was not appeared in table2.

**Answer:**

I’m very sorry for our mistake about Primers IL-17 and GAPDH, and the the primer of ACTIN added into table2.

Those Primers were used in supplementary specification.

Mucin 2 of human (forward:5_-GTCTGCAAGTGCAACACCAG-3)

(reverse: 5_-GCATTCCCGTGAACACACAC -3_).

HES-1 of human (forward:5_-AAAAATTCCTCGTCCCCGGT-3)

(reverse: 5_-ATGCCGCGAGCTATCTTTCT -3_).

MATH-1 of human (forward:5_-CAGCTGCGCAATGTTATCCC -3)

(reverse: 5_-TTGTAGCAGCTCGGACAAGG -3_).

3. Which statistical analysis are used to analyze the data? It not shown in methods.

**Answer:**

I’m very sorry for our mistake about *forgetting submit this part. The* statistical analysis are used to analyze the data as follow.

*2.6 Statistical analysis*

One-way ANOVA was used to analyze the data expressed as mean ± SD for comparison between multiple groups and least significant difference *t*-test for internal group comparison. All statistical analyses were performed using SPSS version 19. The threshold of statistical significance was set to *P* < 0.05. GraphPad Prism version 6.02 was used to generate histograms.

4. From the figure legend, *** mean p<0.001 vs. water group, but why the water group are compared with itself in figure1A? **Besides,** **bodyweight in day5 and day6 as well as** **DAI in day5 are really significant different between control group and QBD group?** In addition, the colons in control group were placed deviously but the colons in QBD group were placed uprightly, which may lead to the error of colon length.

**Answer:**

It is really true as reviewer suggested that we should not make the water group compared with itself in figure1A. we planned to express that the weight of mice in water group was increasing without any treatment, but it was less relation with this topic. Secondly, **the bodyweight in day5 and day6 in QBD group was 96.8±1.08 、94.68±1.97 , the** **bodyweight in day5 and day6 in control group was 92.89±1.26 、89.82±1.40.the DAI in day5 and day6 in QBD group was 1.5±0.75 、2.12±0.99, the DAI in day5 and day6 in control group was 2.25±0.46 、4.5±0.75. The DAI and bodyweight are really significant different between control group and QBD group. Finally, Because the** Mucosal inflammation 、stool mass and Pathological adhesion in colon, which make colons were placed deviously. But he length of each colon in all groups was measured respectivel**y (showed in figure 1B).**

5. Why the Ki67 and Caspase-3 were determined? In Fig2.D it is caspase-3, but not capased-3.

**Answer:**

Under normal conditions epithelial cell shedding does not contribute to an alteration of the intestinal barrier ,However, the increased apoptotic rate of epithelial cell in UC was reported, which may induce a decrease in barrier function ^1、2、3^.There are some evidences show that the apoptotic rate probably related to barrier function^4、5^.Thus, we take caspased-3(The main terminal shearing enzyme in the process of apoptosis) as a maker of apoptosis and Ki67 as a maker of proliferation, which evaluating the treatment effect of QBD on DSS-induced colon epithelial injury and colitis. Secondly, I’m very sorry for our incorrect writing about “caspase-3” and “caspased-3”.

6. In Fig 2E, the protein expression of active Caspase-3 should be change into ratio of cleaved Caspase-3 over Caspase-3.

**Answer:**

**As Reviewer suggested that we make a picture about the** ratio of cleaved Caspase-3 over Caspase-3 as follow.

7. Abbreviation of SASP is commonly considered sulfasalazine but not mesalazine.

**Answer:**

**We have made the correction according to the Reviewer comment. We used** mesalazine group to replace SASP group.

8. In the part of result, many important references were missing and some brief summary are inappropriate. For example, the author considered that QBD prevent the loss of muc-2 expression in DSS mice by inhibiting Notch-signaling probably. This hypothesis should be testified that the effect of QBD on muc-2 is less efficient when Notch-signaling is blocked.

**Answer:**

Thanks for your kindly comments. First, we took corrective action about some inappropriate brief summary. Secondly, after Notch intercellular domain (NICD) translocates into the nucleus, it activates transcription^6^. These events result in transcription of target genes such as HES1 and math-1^7,8^. In previous studies show that differentiation of intestinal stem cells into the goblet cell lineage is facilitated by Math1, which is suppressed by Hes1^9,10,11^. In large intestine, MUC2 is the major secretory mucin of extracellular mucus and secreted by goblet cells^6^. Furthermore, the depletion of goblet cells in Ulcerative Colitis is associated with Suppression of Hath1 Gene Expression Directly Regulated by Hes1 Via Notch Signaling^12,13^. We used the inhibition of NOTCH Signaling (DAPT) in SW620 cells and found it did not significantly modulate ERK1/2 phosphorylation. However, treated with the MEK inhibitor (U0126) play a down - regulation effects of NOTCH signaling significantly in SW480 cells, which is Consistent with previous reports^14,15^(as follow picture B). In vitro experiment, the expression of p-ERK and NICD in SW480 decreased significantly after the treatment of QBD Drug-Containing Serum (15% 12h)^16^ were detected using immunoblotting (as follow picture A). The results were same in our vivo experiment. To further testify QBD prevent the loss of muc-2 expression in DSS mice by inhibiting Notch-signaling probably. Sw480 cells were treated with DAPT (70nM) for 240 mins in DAPT group. In DAPT+QBD group, SW620 cells were pre-treated with DAPT for 240 mins, then removed and replaced by normal culture media containing DAPT (70nM) and QBD Drug-Containing Serum (15%) for 12 h. We found that the mRNA expression of MUC-2 and MATH-1 increased significantly in DAPT group compared with NC group. However, there were no statistically significant differences of the mRNA expression of MUC-2 and MATH-1 between DAPT+QBD group and DAPT group (as follow picture C). Thus, our results suggested that the effect of QBD on muc-2 is less efficient probably due to Notch-signaling is blocked.

**Drug-Containing Serum Preparation**

Twenty-four SD rats were divided into two groups equally: the blank group treated with distilled water, the QBD Drug-Containing Serum group treated with Chinese herbal compound for QBD(0.036ml/g). All treatments were administered once a day for 7 successive days by gastric infusion. Blood of rats was collected 1 h after final administration from abdominal aorta under sterilization, kept unchanged for 1 h, and centrifuged at 3 000 r/min for 15 min to separate the drug-serum. The drug-sera from rats of the same groups were mixed, subjected to 56℃ water bath for 30 min to inactivate, filtrated with microporous membrane to eliminate germs, sub-packaged in tubes, and preserved under -70℃ until ready for testing.

9. ERK doesn’t increased in the control group.

**Answer:**

Considering the Reviewer’s suggestion, we have made a picture about the ratio of P-ERK over total ERK as follow.

**R E FE R E N C E S**

1.Watson AJM, Chu S, Sieck L, *et al.* Epithelial barrier function *in vivo* is sustained despite gaps in epithelial layers. Gastroenterology 2005; 129: 902-12.

2. Schulzke JD, Bojarski C, Zeissig S, Heller F, Bitter AH, Fromm M. Disrupted barrier function through epithelial cell apoptosis. Ann NY Acad Sci 2006; 1072: 288-99.

3. Heller F, Florian P, Bojarski C, *et al.* Interleukin-13 is the key effector Th2 cytokine in ulcerative colitis that affects epithelial tight junctions, apoptosis and cell restitution. Gastroenterology

2005; 129: 550-64.

4.Zeissig S, Bojarski C, Buergel N, *et al.* Downregulatuion of epithelial apoptosis and barrier repair in active Crohn’s disease by tumor necrosis factor _ antibody treatment. Gut 2004; 53: 1295-302.

5. Kiesslich R, Duckworth CA, Moussata D, *et al.* Local barrier dysfunction identified by confocal laser endomicroscopy predicts relapse in inflammatory bowel disease. Gut 2012; 61: 1146-53.

6.Noah T K, Shroyer N F. Notch in the intestine: regulation of homeostasis and pathogenesis[J]. Annual Review of P-===hysiology, 2013, 75(1):263-288.

7. Jensen J, Pedersen EE, Galante P, Hald J, Heller RS, et al. 2000. Control of endodermal endocrine development by Hes-1. *Nat. Genet.* 24:36–44

8. Suzuki K, Fukui H, Kayahara T, Sawada M, Seno H, et al. 2005. Hes1-deficient mice show precocious differentiation of Paneth cells in the small intestine. *Biochem. Biophys. Res. Commun.* 328:348–52

9 Yang Q, Bermingham NA, Finegold MJ, Zoghbi HY. RequireMent of Math1 for secretory cell lineage commitment in the mouse intestine. Science. 2001;294:2155?8.

10. Kageyama R, Ohtsuka T, Tomita K. The bHLH gene Hes1 regulates differentiation of multiple cell types. Mol Cells.2000;10:1–7

11. Shroyer NF, Helmrath MA, Wang VY, Antalffy B, Henning SJ,Zoghbi HY. Intestine-specific ablation of mouse atonal homolog1 (Math1) reveals a role in cellular homeostasis. Gastroenterology.2007;132:2478?88.

12 Fre S, Huyghe M, Mourikis P, et al. Notch signals control the fate of immature progenitor cells in the intestine. Nature. 2005;435:964–968.

13. Zheng X, Tsuchiya K, Okamoto R, et al. Suppression of hath1 gene expression directly regulated by hes1 via notch signaling is associated with goblet cell depletion in ulcerative colitis[J]. Inflammatory Bowel Diseases, 2011, 17(11):2251-2260.

14. Pope J L, Bhat A A, Sharma A, et al. Claudin-1 regulates intestinal epithelial homeostasis through the modulation of Notch-signalling[J]. Gut, 2014, 63(4):622-634.

15. Tremblay I, Paré E, Arsenault D, et al. The MEK/ERK pathway promotes NOTCH signalling in pancreatic cancer cells.[J]. Plos One, 2013, 8(12):e85502.

16蒋跃绒, 缪宇, 杨琳,等. Effect of Chinese Herbal Drug-Containing Serum for Activating-Blood and Dispelling-Toxin on ox-LDL-Induced Inflammatory Factors'Expression in Endothelial Cells[J]. Chinese Journal of Integrative Medicine, 2012, 18(1):30-33.
